# Finishing Barrow Skeletal Muscle Performance and Fatigue Response to Large-Dose Nicotinamide Riboside Supplementation

**DOI:** 10.3390/metabo16040261

**Published:** 2026-04-13

**Authors:** Daniela A. Alambarrio, Xiaohan Li, Siara S. Zedonek, Sophia E. Willis, Jordan N. Proctor, Faezeh Mozafari, Jarrod A. Call, Litzy E. Delgado, McKenna S. Doran, John M. Gonzalez

**Affiliations:** 1 Department of Animal Science, University of Georgia, Athens, GA 30602, USA; alambarriod@uga.edu (D.A.A.); xiaohan.li96@uga.edu (X.L.); siara.zedonek@uga.edu (S.S.Z.); sophia.willis@uga.edu (S.E.W.); jordan.proctor1@uga.edu (J.N.P.); litzy.delgado@uga.edu (L.E.D.); mckenna.doran@uga.edu (M.S.D.); 2 Department of Physiology and Pharmacology, College of Veterinary Medicine, University of Georgia, Athens, GA 30602, USA; faezeh.mozafari@uga.edu (F.M.); call@uga.edu (J.A.C.); 3 Regenerative Bioscience Center, University of Georgia, Athens, GA 30602, USA

**Keywords:** electromyography, mitochondrial activity, muscle performance, vitamin B3

## Abstract

**Background/Objective:** Delaying muscle fatigue could alleviate economic and food security, and welfare concerns associated with transporting market-weight pigs to harvest. Previous research demonstrates barrow nicotinamide riboside (**NR**) supplementation at varying doses during the last 10 d of finishing shows to be a countermeasure to muscle fatigue by reducing muscle fiber recruitment and increasing mitochondrial DNA expression in a dose-dependent manner. Therefore, this study aims to determine if a greater NR dose further enhances barrow fatigue resistance and characterize muscle mitochondria content and efficiency. **Methods:** Barrows (*N* = 87) were assigned to one of two dietary NR supplementation doses (**TRT**): 0 (**0NR**) or 150 (**150NR**) mg/kg body weigh NR administered during the last 14 d of finishing. Muscle (**MUS**) biopsies were collected on supplementation d (**DAY**) 0, 7 and 14 from three hind-leg muscles for NAD+ quantification and mitochondrial DNA expression and efficiency. On days 15 and 16, barrows were subjected to a performance test until they were subjectively exhausted. Electromyography data collection during the performance test were divided into five periods (**PER**) and included normalized root mean square (**nRMS**) from the same muscles. **Results:** There were no three-way interaction for nRMS (*p* > 0.83), but there were MUS × TRT and PER × TRT interactions (*p* < 0.05). During performance testing, 150NR had greater nRMS than 0NR in the *bicep femoris* (**BF**) and *tensor fasciae latae* (**TFL**; *p* < 0.01), but there were no differences in the semitendinosus (**ST**; *p* = 0.77). Treatments did not differ during PER 1 and 2 (*p* > 0.14) but 150NR had greater nRMS than 0NR during PER 3, 4 and 5 (*p* < 0.01) across all muscles. There was no three-way interaction for normalized (**nNAD+**; *p* = 0.14), but there was a DAY × TRT interaction (*p* < 0.05). There were no differences between 0NR and 150NR at d 0 (*p* = 0.95); however, by d 7 and 14, 150NR muscles had greater nNAD+ than 0NR muscles (*p* < 0.01). There tended to be a three-way interaction for mitochondrial DNA expression (*p* = 0.09). At supplementation d 14, all 150NR muscles had greater mitochondrial DNA expression and electron transport chain complex I and II activities (*p* < 0.01). When normalized to citrate synthase activity, electron transport chain complex I and II activity did not differ (*p* > 0.05). **Conclusions:** Large-dose NR supplementation appears to support sustained muscle fiber recruitment during prolonged activity and enhance fatigue resilience, primarily through increased NAD+ and mitochondrial biomarkers abundance and not through mitochondrial efficiency.

## 1. Introduction

The term “fatigued pig syndrome” was introduced by the pig industry to describe fatigue induced by transport, and these pigs are often categorized as non-ambulatory when describing transport losses [1]. Fatigued pigs typically show no physical injury but exhibit clear signs of distress such as open-mouth breathing, skin discoloration, muscle tremors, and reluctance to walk or keep pace with the group [2]. Non-ambulatory pigs represent a substantial economic loss, with discounts of up to 30% of the pig’s total value in the U.S. industry [3]. Although economic estimates from other countries are limited, as stated by Ritter et al. [3], non-ambulatory pigs cost the U.S. approximately $37 million annually, equivalent to the loss of 164 million 0.11 kg servings of boneless pork [4]. Despite available literature describing fatigue responses to various stressors, there remains limited information on biological stress inducers and underlying mechanisms that contribute to fatigued pig syndrome.

Pyridine nucleotides, such as nicotinamide adenine dinucleotide (**NAD+**), serve as essential muscle metabolism co-substrates and play a role in fatigue onset [5]. As a redox coenzyme, NAD+ is reduced to NADH to shuttle electrons to the electron transport chain (**ETC**) for aerobic ATP synthesis. Alternatively, during anaerobic metabolism, the reduction in pyruvate to lactate by lactate dehydrogenase is essential for regenerating the NAD+ pool, thereby maintaining the glycolytic flux necessary for continued ATP production [6]. Rapid NAD+ turnover or depletion is associated with reduced intracellular ATP availability [7]. Isolated NAD+ disturbances are uncommon in biology; however, ATP impairment or limited production are mainly hypothesized to catalyze muscle fatigue [6,8]. Aside from cellular homeostasis during energy metabolism for muscle contraction, NAD+ participates in several regulatory pathways involving lifespan, mitochondrial function, apoptosis, and telomere maintenance [9,10,11]. Specifically, elevated NAD+ concentration stimulates SIRT1 deacetylation of signaling proteins such as PGC-1α, which activates the nuclear respiratory factor and mitochondrial transcription factor A, both key mitochondrial DNA transcription and replication regulators [12,13,14]. Therefore, nutritional strategies aimed at increasing NAD+ bioavailability are considered to support muscle energetics [1,14,15].

Nicotinamide riboside (**NR**) is a naturally occurring pyridine nucleoside and vitamin B3 analog that contributes to NAD+ levels in different mice and human’s muscle groups [14,16]. Studies indicate NR is more orally bioavailable and a more potent NAD+ booster than other B3 vitamins such as nicotinamide and nicotinic acid [17]. Specifically, NR is well tolerated at large clinical doses (up to 3000 mg/d), whereas painful skin flushing and sirtuin inhibition are associated with nicotinic acids and large-dose nicotinamide [17,18]. In mammals, NR serves as a salvageable precursor for NAD+ and is converted to NAD+ through the short, 2- to 3-step salvage pathway [19]. This pathway is more efficient than the multi-step biosynthetic sequences of the Preiss–Handler pathway and the tryptophan-mediated de novo pathway, both of which require several intermediate conversions before contributing to NAD+ pools [1,20]. In finishing barrows, dietary NR inclusion at doses up to 45 mg/kg body weight^−1^·d^−1^ delayed fatigue onset, increased active muscle fibers at fatigue and reduced fatigue-associated metabolic markers [21]. In the same study, NR supplementation increased mitochondrial DNA expression in a muscle- and dose-dependent manner; however, this increase did not clearly translate to a linear improvement in fatigue resistance across all doses, possibly due to issues with barrows consuming their calculated dose or the dose being insufficient to reach critical biological thresholds. Conze et al. [22] reported a dose-dependent increase in NAD^+^ concentrations in human urine and blood following NR supplementation, with greater supplementation doses eliciting greater NAD+ increases, suggesting the upper limit of efficacy has not yet been reached in large animal models. While previous porcine utilized doses up to 45 mg/kg, robust performance outcomes in mouse studies required dosages exceeding 100 mg/kg [14]. Therefore, the objective of this study is to determine whether a larger NR dose elevates NAD+ levels and determine if these increased levels induce greater improvements in barrow fatigue resistance, muscle fiber type composition, oxidative activity, and mitochondrial DNA expression.

## 2. Materials and Methods

The University of Georgia Institutional Animal Care and Use Committee approved the protocol used in this experiment, A2023 05-005-Y3-A3. Sample size was calculated using data generated by Henessy et al. [21] to detect a 33% difference in muscle RMS at an α of 0.05 and β of 0.20. The predicted variation used was 1.31 m/s^2^. More animals than calculated were utilized because past studies utilizing the performance test model explained below reveal some pigs would have to be excluded due to behavior issues.

### 2.1. Live Animal Management

Over five replicates, finishing barrows (*N* = 87; Camborough × PIC 337; Pig Improvement Company, Hendersonville, TN, USA) were individually housed in an environmentally controlled room at the University of Georgia Large Animal Research Unit (**LARU**; Athens, GA, USA). Barrows were transported to LARU from the University of Georgia Swine Unit (Winterville, GA, USA) on d 0 of the trial; barrows were stratified by weight and randomly assigned one of two dietary treatments (**TRT**) in a 1:1 ratio within each replicate. Three replicates consisted of 20 barrows, one of 21 barrows and another one of 6 barrows. The replicate with 21 barrows consisted of 11 0NR barrows and 10 150NR. Barrows were housed in pens equipped with a 2-hole dry feeder (Farmweld, Teatopolis, IL, USA) and nipple waterer to allow for *ad libitum* access to feed and water. After a 7 d acclimation period, barrows were subjected to their dietary treatment of the basal diet supplemented with 0 (**0NR**, *n* = 44) or 150 (**150NR**, *n* = 43) mg·kg body weight^−1^·d^−1^ NR (Niagen Biosciences; Los Angeles, CA, USA). Body weights and feed consumption were recorded weekly to determine average daily feed intake to calculate each barrow’s NR supplementation rate. Weekly recalibrations maintained the target mg/kg dose relative to individual growth and daily intake fluctuations.

### 2.2. Muscle Biopsy Collection

The methods of Hennesey et al. [21] were followed with slight modifications. On supplementation d 0, 7, and 14, the *bicep femoris* (**BF**), *semitendinosus* (**ST**), and *tensor fasciae latae* (**TFL**) muscles were biopsied for muscle metabolite analysis. After site preparation, including cleaning and local anesthesia administration, muscle biopsies were obtained from the right hindlimb on d 0 and 14, whereas biopsies on d 7 were taken from the left hindlimb to avoid regenerating or previously damaged muscle tissue. Roughly 100 mg of tissue were collected from each muscle site and partitioned into individual 2 mL centrifuge tubes corresponding to the specific metabolite assay. All samples were rapidly frozen by submersion in liquid nitrogen and subsequently stored at −80 °C until analysis.

### 2.3. Performance Test

On supplementation d 15 for reps 1 to 3 and d 16 for reps 4 to 5, barrows were randomly assigned to an individual performance test order. Prior to performance testing, the barrow’s hind left limb was prepared for EMG electrodes (Noraxon, Scottsdale, AZ, USA) attachment as stated by Hennesey et al. [21].

During performance testing, barrows were briskly walked around a 270 m course by two handlers until subjective fatigue was reached. Subjective fatigue was determined by handlers when barrows stopped five times, refused to walk after encouragement, and displayed physical fatigue signs such as open-mouth heavy breathing, wheezing, skin discoloration, or muscle tremors. Performance data collected included total exhaustion time, distance covered and speed. If barrows spent more than 25% of their total performance test resisting handlers or veering off course, they were excluded from analyses (0NR *n* = 1; 150NR *n* = 5). After performance testing, barrows were returned to their corresponding pens and recovered for one to two d according to their respective replication number. On d 17, barrows were loaded into a trailer and transported to the University of Georgia Meat Science and Technology Center (Athens, GA, USA) for harvest using an USDA-approved protocol and stored for 24 h postmortem chilling.

### 2.4. Electromyography Analysis

The methods of Hennessey et al. [21]. were followed with slight modifications for EMG analysis using a custom program using MATLAB R2024b (MathWorks, Inc., Natick, MA, USA). Each barrow’s raw EMG data were analyzed for each electrical burst corresponding to each muscle contraction during performance testing by using a 6th order Butterworth filter. The EMG frequency was considered as median power frequency (**MdPF**), while amplitude was considered as root mean square (**RMS**). Data were averaged into five periods during the performance test and individually normalized (**nMdPF**; **nRMS**) to the first 30 s of each barrow’s test. Normalized values greater than 200% were classified as non-physiological mechanical artifacts and added as missing values for statistical analyses, as those values indicate vigorous sensor movement caused transient sensor–skin separation (0NR *n* = 212 across all three muscles and five time periods; 150NR *n* = 227 across all three muscles and five time periods).

### 2.5. Immunohistochemistry and Histochemistry

During carcasses fabrication on 24 h postmortem, a one cm^3^ portion of each muscle located where the EMG sensor was placed was collected, embedded in optimal cutting temperature (Neg-50, Epredia, Kalamazoo, MI, USA) and tissue-freezing medium, frozen with liquid nitrogen-cooled methyl butane, and stored at −80 °C until further processing. Three slides with two 10 μm thick cryosections per slide were collected from each muscle on positively charged slides (Midsci, Fenton, MO, USA) for fiber type, succinate dehydrogenase (**SDH**) staining and capillary stanning.

The methods of Paulk et al. [23]. were followed for fiber type immunohistochemistry with modifications. Cryosections were incubated with 5% horse serum and 0.2% Triton-X in PBS for 30 min to block all non-specific binding antigens. Cryosections were incubated overnight at room temperature with a primary antibody cocktail consisting of a blocking solution and 1:100 supernatant myosin heavy chain type I, IgG2b (BAD5; Developmental Studies Hybridoma Bank, University of Iowa, Iowa City, IA, USA), 1:100 supernatant myosin heavy chain type IIA, IgG1 (SC-71: Developmental Studies Hybridoma Bank), and 1:100 supernatant myosin heavy chain type IIB, IgM (BF-F3; Developmental Studies Hybridoma Bank). Cryosections were washed with PBS three times for 5 min and incubated for 1 h with a secondary antibody cocktail containing a blocking solution and 1:1000 Alexa-Fluor 488 goat anti-mouse IgM, Alexa Fluor 594 goat anti-mouse IgG1, Alexa Fluor 633 goat anti-mouse IgG2b, and wheat germ antigen (**WGA**) Alexa Fluor 594. Sections were washed with PBS three times for 5 min, covered with 9:1 glycerol, and cover-slipped for imaging.

The methods of Noel et al. [24] 2016 were followed for SDH staining with slide modifications. Cryosections were incubated for 1.5 h in prewarmed solutions. After incubation, slides were washed 3 times for 1 min, covered with 5 μL of 9:1 glycerol and cover-slipped for imaging.

Capillary density sections were blocked using the same solution utilized for fiber type immunohistochemistry. Sections were incubated overnight with a primary antibody cocktail, containing 1:50 α-CD-31 Pecam-1 antibody (Invitrogen) in blocking solution. Sections were washed three times for 5 min with PBS and incubated for 30 min with a secondary antibody cocktail containing 1:1000 Alexa-Flour 488 goat-anti-rabbit heavy and light chains (Invitrogen) and WGA in a blocking solution. Sections were washed with PBS three times for 5 min, covered with 5 μL of 9:1 glycerol and cover-slipped for imaging.

All cryosections were imaged at 40× magnification using a Revolve 4 Upright, Inverted, Brightfield, Fluorescent Microscope (ECHO Laboratories; Radnor, PA, USA). Immunohistochemistry cross-sectional area (**CSA**) and percentage data were obtained by tracing the area within the WGA perimeter per fiber type. Fiber types were determined based on color under fluorescence, were fibers that stained positive for BAD-5, SC-71 and BF-F3 were considered type I, type IIA, and type IIB respectively. Fibers that simultaneously stained for SC-71 and BF-F3 were considered type IIX. Capillary density was calculated by dividing the capillary number by the fiber number within a photomicrograph. A minimum of 500 fibers were measured for fiber typing, CSA, and capillary density with their corresponding capillaries. During SDH intensity analysis, the same fibers and their corresponding fiber types identified by immunohistochemistry were located in the cryosections, and the mean stain intensity was quantified using ImageJ v.1.54r software (National Institutes of Health, Bethesda, MD, USA).

### 2.6. NAD+ Quantification

Nicotinamide adenine dinucleotide muscle content was determined from 20 mg muscle biopsies collected on d 0, 7, and 14. Levels of NAD+ were determined following the NAD/NADH Assay Kit manufacturer instructions (Colorimetry; Abcam, Cambridge, UK). Sample tissue was homogenized using a Dounce homogenizer with approximately 45 passages with extraction buffer and centrifuged at high speed. Supernatant was filtered using amicon ultra centrifugal filter 10 kDa (Millipore Sigma, Darmstadt, Germany). The filtrated supernatant was plated with reaction mix and developer. Nicotinamide adenine dinucleotide levels were read using a spectrophotometer (Biotek, Winooski, VT, USA) after one hr of incubation at 450 nm. Data were normalized (**nNAD+**) to each muscle’s 0NR NAD+ on d 0.

### 2.7. Mitochondrial DNA Expression

Total deoxyribonucleic acids (**DNA**) were extracted from 25 mg of tissue collected on d 0 and 14 of the trial by following the manufacturer’s instructions of the Qiagen DNeasy Blood & Tissue Kit (Qiagen, Germantown, MD, USA). In duplicates, 10 ng of total DNA were amplified using TaqMan Master Mix (Applied Biosystems, Foster City, CA, USA). The forward, reverse primers and thermocycler parameters were utilized according to Hennesey et al. [21] and gene fold-change expression levels were calculated following Livak and Schmittgen [25]. Expression fold was normalized to beta actin expression (∆Ct) and calibrated to each muscle’s 0NR barrows’ mitochondrial D-loop expression on d 0 (∆∆Ct).

### 2.8. Mitochondrial Enzyme Activity

Tissues from each muscle biopsy collected on d 0 and 14 were homogenized (Omni Tissue Homogenizer, Kennesaw, GA, USA) in 400 μL of 0.33 M phosphate buffer (pH 7.4). Citrate synthase (**CS**) and electron transport chain complexes I (**ETCI**) and II (**ETCII**) activity were determined by using a spectrophotometer (Molecular Devises, San Jose, CA, USA) following the methods of Heo et al. [26]. Citrate synthase activity was monitored by the reduction of 5′,5′-dithiobis 2-nitrobenzoic acid over time by measuring absorbance at 412 nm. Complex I activity was determined by measuring the oxidation of **NADH** via 2,6-Dichlorophenolindophenol (**DCPIP**) reduction at 600 nm. Complex II activity was determined by initiating the assay with oxidized decyl-ubiquinone and reduction in DCPIP at 600 nm. Data were normalized (**nETCI**; **nETCII**; **nCS**) to each muscle’s 0NR enzyme activity on d 0.

### 2.9. Statistics

Performance test data were analyzed as randomized incomplete block design with the barrow as the experimental unit. The TRT served as the fixed effect and repetition served as the random effect. Electromyography, NAD+, mitochondrial DNA expression, and mitochondrial enzyme activity data were analyzed as randomized incomplete block split-plot design with repeated measures. The barrow served as the whole-plot and the muscle (**MUS**) served as the sub-plot. Fixed effects included TRT, MUS, biopsy collection day (**DAY**) or period (**PER**), and their two- and three-way interactions. Random effects were repetition and DAY or PER served as the repeated measure with barrow within TRT as the subject and compound of symmetry as the covariance structure. Muscle histological morphometrics data were analyzed as randomized incomplete block split-plot design with the barrow as the whole-plot and MUS as the sub-plot. Fixed effects included TRT, MUS, and their interaction. The random effect was repetition. All data were analyzed using SAS 9.3 (SAS Institute Inc., Cary, NC, USA). Pairwise comparisons between least square means of the factor level comparisons were computed using the PDIFF option of the LSMEANS statement. Statistical significance was determined at *p* < 0.05 and trends at 0.05 < *p* ≤ 0.10.

## 3. Results

### 3.1. Performance Test

There were no TRT effects for lap number, distance, time and speed (*p* > 0.60; Table 1).

### 3.2. Electromyography

**Figure 1:**
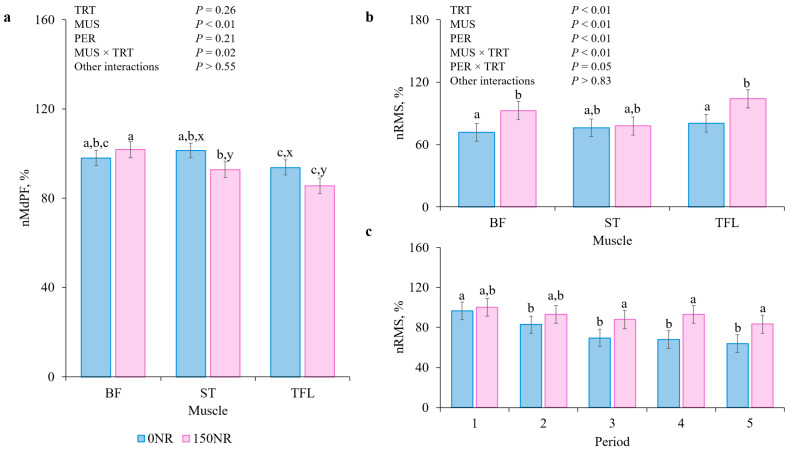
Effects of large-dose nicotinamide riboside supplementation on electromyography-derived measures of *bicep femoris* (**BF**), *semitendinosus* (**ST**), and *tensor fasciae latae* (**TFL**). (**a**) Median power frequency (**MdPF**) and (**b**,**c**) root mean square (**RMS**). Data were averaged into five periods during the performance test and individually normalized (**nMdPF**; **nRMS**) to the first 30 s of each barrow’s test. Error bars indicate ± SEM (*n* = 43 0NR; *n* = 38 150NR). ^a,b,c^ Means within a panel with different superscripts differ (*p* < 0.05). ^x,y^ Means within a panel with different subscripts tended to differ (0.05 < *p* ≤ 0.10).

There were no TRT × MUS × PER, MUS × PER or PER × TRT interactions for nMdPF (*p* > 0.55) but there was a MUS × TRT interaction (*p* = 0.02; Figure 1). In the BF, 0NR did not differ from 150NR nMdPF (*p* = 0.53), while 0NR ST and TFL tended to have greater nMdPF than 150NR (*p* < 0.09).

There were no MUS × TRT × PER, and MUS × PER interactions for nRMS (*p* > 0.83) but there were MUS × TRT and PER × TRT interactions (*p* < 0.05). Over the entire performance test, 150NR BF and TFL had greater nRMS than 0NR (*p* < 0.01), while there was no difference (*p* = 0.77) in the ST. Across all muscles, there were no TRT differences during periods 1 and 2 (*p* > 0.14) but for PER 3, 4 and 5, 150NR muscles had greater nRMS than 0 NR muscles (*p* < 0.01).

### 3.3. Immunohistochemistry and Histochemistry

There were MUS × TRT interactions for all muscle characteristics (*p* < 0.01) except there was a tendency for type I fiber SDH intensity interaction (*p* = 0.08; Table 2).

In all muscles, 0 NR barrows had greater type I fiber percentage than 150NR barrows (*p* < 0.01). In the BF, 0NR barrows had greater (*p* < 0.01) type IIA fiber percentage than 150NR barrows, 150NR barrows had greater (*p* < 0.01) type IIA percentage than 0NR barrows in the TFL, and there were no differences (*p* = 0.31) in the ST. For type IIX fibers, 0NR barrows had a smaller (*p* < 0.01) percentage than 150NR barrows in the BF, while 150NR barrows had smaller ST and TFL type IIX percentages than 0NR barrows (*p* < 0.01). In all muscles, 0NR barrows had smaller type IIB percentages than 150NR barrows (*p* < 0.05).

Control barrows had greater type I, IIA and IIB fiber CSA than 150NR barrows in the BF (*p* < 0.01), while 0NR barrows had smaller CSA for the same fibers in the ST and TFL (*p* < 0.01). Control barrows had smaller type IIX CSA than 150NR barrows in the BF and TFL (*p* < 0.01), but had bigger CSA in the ST (*p* < 0.01).

Control barrows had greater (*p* = 0.05) BF type I fiber SDH intensity than 150NR barrows, but there were no ST and TFL differences (*p* > 0.15). There were no differences in BF and TFL type IIA SDH intensity (*p* > 0.17), while 0NR barrows had greater (*p* < 0.01) ST SDH intensity than 150NR barrows. Control barrows had smaller BF type IIX and IIB SDH intensities than 150NR barrows (*p* < 0.04), 150NR barrows had smaller ST type IIX and IIB intensities than 0NR barrows (*p* < 0.01), and there were no TFL type IIX and IIB intensity differences (*p* > 0.46).

Control barrows has a smaller (*p* = 0.05) capillary ratio than 150NR barrows in the BF while having a greater (*p* = 0.05) ratio in the TFL, and no differences (*p* = 0.55) in the ST.

### 3.4. NAD Quantification

**Figure 2:**
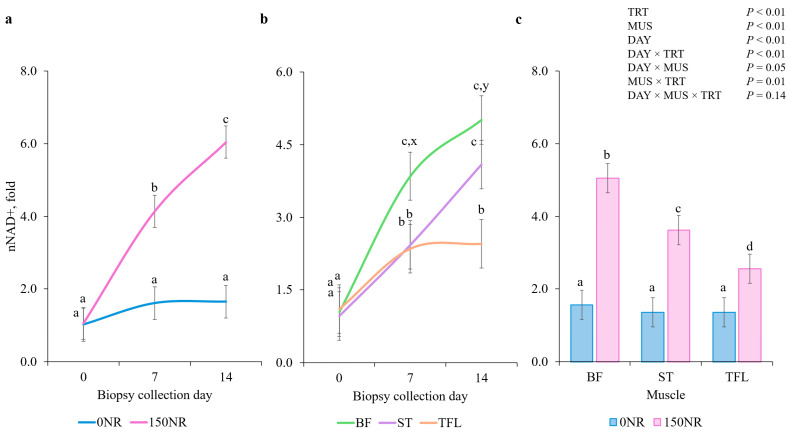
(**a**) Day × treatment (**TRT**), (**b**) day × muscle (**MUS**), and (**c**) MUS × TRT interactions for normalized nicotinamide adenine dinucleotide (**nNAD+**) when a large dose of nicotinamide riboside was administered to barrows during the last 14 d of feeding. Barrows were supplemented on 0 (**0NR**) or 150 (**150NR**) mg·kg body weight^−1^·d^−1^ nicotinamide riboside during the last 14 d of feeding. Data are normalized to d 0 0NR NAD+ within each muscle (*bicep femoris* (**BF**); *semitendinosus* (**ST**), *tensor fasciae latae* (**TFL**). ^a,b,c,d^ Means within different superscripts differ (*p* < 0.05). Error bars indicate ± SEM (*n* = 44 0NR; *n* = 43 150NR). ^x,y^ Means with different subscripts tend to differ (0.05 < *p* ≤ 0.10).

There were no DAY × MUS × TRT interactions for nNAD+ (*p* = 0.14; Figure 2), but there were DAY × TRT, DAY × MUS and MUS × TRT interactions (*p* < 0.05). Treatments did not differ at d 0 (*p* = 0.95); however, by d 7 and 14, 150NR muscles had greater nNAD+ than 0NR muscles (*p* < 0.01). On d 0, muscles did not differ in nNAD+ (*p* > 0.83) but by d 7, BF had greater nNAD+ than ST and TFL (*p* < 0.01), which did not differ (*p* = 0.90) from each other. On d 14, BF and ST had greater nNAD+ than TFL (*p* < 0.01), and BF and ST were not different (*p* = 0.16) from each other. Across all days, all 150NR muscles had greater nNAD+ than 0NR muscles (*p* < 0.01).

### 3.5. Mitochondrial DNA Expresssion

**Figure 3:**
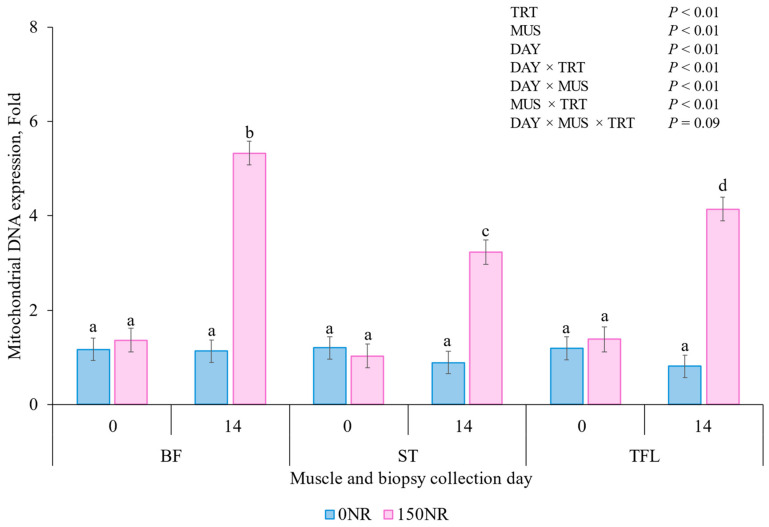
Effects of large-dose nicotinamide riboside on supplementation d 0 and 14 *bicep femoris* (**BF**); *semitendinosus* (**ST**), *tensor fasciae latae* (**TFL**) mitochondrial DNA expression. Barrows were supplemented on 0 (**0NR**) or 150 (**150NR**) mg·kg body weight^−1^·d^−1^ nicotinamide riboside during the last 14 d of feeding. Error bars indicate ± SEM (*n* = 44 0NR; *n* = 43 150NR). ^a,b,c,d^ Means with different superscripts differ (*p* < 0.05).

There tended to be a DAY × MUS × TRT interaction for muscle mitochondrial DNA expression (*p* = 0.09; Figure 3). On d 0 and for all muscles, mitochondrial DNA expression did not differ between 0NR and 150NR muscles (*p* > 0.11); however, on d 14 and for all muscles, 150NR muscles had greater expression than 0NR muscles (*p* < 0.01).

### 3.6. Mitochondrial Enzyme Activity

**Figure 4:**
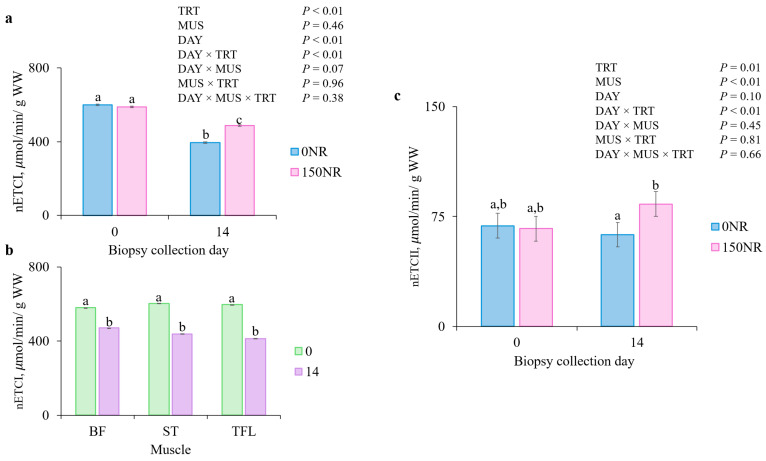
(**a**) Normalized electron transport chain complex I activity (**nETCI**) day × treatment (**TRT**) and (**b**) day × muscle (**MUS**) interactions, and normalized electron transport chain complex II activity (**nETCII**). (**c**) Day × TRT interaction when large dose nicotinamide riboside was supplemented during the last 14 d of feeding. Barrows were supplemented on 0 (**0NR**) or 150 (**150NR**) mg·kg body weight^−1^·d^−1^ nicotinamide riboside during the last 14 d of feeding. Data are normalized to d 0 0NR enzyme activity within each muscle (*bicep femoris* [**BF**]; *semitendinosus* [**ST**], *tensor fasciae latae* [**TFL**]) of each assay. Error bars indicate ± SEM (*n* = 44 0NR; *n* = 43 150NR). ^a,b,c^ Means within a panel with different superscripts differ (*p* < 0.05).

There were no DAY × MUS × TRT or MUS × TRT interactions for nETCI or nETCII (*p* > 0.38; Figure 4). There were DAY × TRT interactions for nETCI and nETCII (*p* < 0.01) and there tended to be a DAY × MUS interaction (*p* = 0.07) for nETCI but not for nETCII (*p* = 0.45). On d 0, 0NR and 150NR nETCI did not differ (*p* = 0.62); however, on d 14, 150NR had greater (*p* < 0.01) nETCI than 0NR. Activity of nETCI decreased from d 0 to d 14 for all muscles (*p* < 0.01). There was no difference (*p* < 0.17) between 0 and 150NR nETCII, but by d 14, 150NR muscles had greater (*p* < 0.01) nETCII than 0NR muscles.

There were no interactions for CS activity (*p* > 0.45; Table 3), but there were DAY, TRT and MUS main effects (*p* < 0.04; Table 3). Activity of nCS was greater for 150NR than 0NR (*p* < 0.01). Bicep femoris tended to have greater nCS than ST (*p* = 0.06) but smaller activity than TFL (*p* < 0.01), while TFL and ST did not differ (*p* = 0.14). Activity of nCS was greater (*p* = 0.03) on d 14 compared to d 0. Mitochondrial enzyme activity was also normalized to CS values; however, there were no interactions observed (*p* > 0.05; Supplementary Table S1).

## 4. Discussion

At commercial abattoirs, many fatigued pigs have recovered within two to three hours after transportation [27], yet many die before they regain the ability to walk independently [2]. Non-ambulatory pigs present economic and welfare challenges to the swine industry; consequently, the literature has focused on fatigue pig syndrome causes and management strategies to reduce muscle fatigue incidence [28]. Nicotinamide adenine dinucleotide is a major cofactor for mitochondrial function, and reduced levels of impaired muscle performance [6,8]. Nicotinamide riboside supplementation increased NAD+ pools and supported skeletal muscle redox balance in different mice models [11,12,14]. Belenky et al. [9] and Berger et al. [10] established the foundational understanding of NR as a potent NAD+ precursor that activates sirtuin-mediated signaling which provides neuroprotective and health-span-extending benefits, and more relevant for this study, supports mitochondrial bioenergetics. Hennesey et al. [21] report supplementing NR at 15 and 30 mg/kg body weight in feed and 45 mg/kg body weight via drench increased movement distance between 27 and 42% compared to non-supplemented barrows. In the same study, barrows receiving the drench dose walked for the longest duration, indicating administration route may affect NR ability to extend performance. At a greater dose of 400 mg/kg body weight, Canto et al. [14] and Zhang et al. [16] report mice ran 30 and 33% longer and farther, respectively, on a motorized treadmill. In the current study, large-dose NR supplementation did not affect barrow performance. This finding may be due to study barrows frequently becoming aggressive toward handlers and often refusing to cooperate with positioning or movement cues during performance testing. Repeated behavioral interruptions limited progressive data collection and probably caused distance and time datasets to be heavily influenced by barrow attitude and resistance to moving around the track. Although similar challenges were reported by Hennesey et al. [21], inherent differences between commercial crossbreeds may have influenced responsiveness to both performance and NR supplementation; however, no research has compared behavioral traits between the crosses. While performance data indicate NR had no effect on performance test fatigue measures, EMG data suggests that NR-supplemented pigs utilized more efficient neuromuscular recruitment to meet performance demands.

Root mean square reflects amplitude changes in the EMG signal over time and is influenced by muscle biomechanical properties and contraction energetic demands [19]. Therefore, RMS serves as a motor unit recruitment indicator and muscle-fiber-firing behavior measure [29]. During repetitive contractions, RMS initially increases as fibers are recruited, followed by a decline as fiber becomes exhausted [30,31]. Treatment-induced nRMS differences are defined by the muscle fatigue curve stage captured during performance testing. In the current study, NR increased BF and TFL nRMS values by 29% over the entire performance period. Within performance periods, NR sustained all muscles’ nRMS across the performance test with increases of 26, 37, and 31% during periods 3, 4, and 5, respectively. These observations contrast Hennessey et al. [21], who reported NR-supplemented barrows had a 65% reduction in nRMS during performance testing. The authors consider this a positive outcome because muscles were still recruiting fibers during the last analysis period, indicating NR barrows were less fatigued. In the current study, 0NR muscles exhibited a nRMS progressive decline, indicating failure to maintain fiber recruitment and fatigue onset, while 150NR muscles maintained a stable fiber activation pattern. The ability of 150NR barrows to maintain greater nRMS suggests that barrows avoided neural decline observed in the 0NR, potentially indicating better-sustain central drive. This interpretation is supported by Chang et al. [32] who suggest that the decline in RMS during sustained contractions represents a central drive failure; conversely, the greater nRMS observed in 150NR barrows indicates a preserved neural strategy which effectively avoids central fatigue. Therefore, the current study’s greater nRMS values indicate NR’s ability to reduce fatigue onset by maintaining more active fibers during fatigue while appearing less susceptible to the cumulative physical demands of the trial. While performance test outcomes reflect the final mechanical output, EMG-derived data provide a physiological window into the underlying neuromuscular strategies and motor-unit recruitment patterns to sustain that output before mechanical failure occurs [32,33].

Force required for pigs to walk during the performance test depends on the contractile mechanism supported by neural, vascular, and metabolic systems, all of which influence muscle fatigue [34]. Sustained contractions alter muscle metabolism and impair action potential propagation, producing progressive muscle conduction velocity decline [35]. The velocity at which action potentials travel along the muscle fiber is represented by the MdPF which typically decreases as contraction repetition continues [36]. In the current study, NR decreased nMdPF values by 4 and 8% in the ST and TFL, respectively. Cockram et al. [37] also observed a decrease in sheep ST when exposed to prolong walking but reported no changes in the TFL. In barrows subjected to performance testing after ractopamine-HCl supplementation, Noel et al. [24] observed no differences in ST and TFL endpoint nMdPF. Observing the same muscles, Hennesey et al. [21] observed supplementing 45 mg/kg of body weight NR by drench increased ST and TFL nMdPF by 15 and 11%, respectively, but NR did not affect activity when supplemented in the feed. Differences among the studies may reflect variations in dose, administration route and may also reflect commercial-barrow muscle physiological, metabolic, and structural characteristic differences. In the current study, reduced nMdPF values were observed from NR muscles at the beginning of performance testing and persisted throughout the entire test. Reduction in nMdPF can arise not only from a fatigue-related process but also from treatment-dependent alterations in baseline neuromuscular activation, membrane excitability or motor-unit recruitment strategy [38,39].

Kupa et al. [40] established muscle fibers’ physical and metabolic properties are the primary EMG power spectrum determinants during fatigue. Over all muscles and barrows, there were 9% type I, 26% type IIA, 12% type IIX, and 53% type IIB muscle fibers, which displayed fewer type I and more type IIA fibers than in Hennesey et al.’s [21] and Noel et al.’s work [24]. While Hennesey et al. [21] reported no NR effect on BF and ST fiber type distribution, the findings of the current study indicate a larger dose provided four days longer elicited a greater alteration in muscle fiber characteristics. The extended supplementation period of this study likely provided sufficient time to capture the remodeling process not evident at 10 d, allowing muscles to move past the initial transient matrix phase, and may have contributed to hypertrophic responses, including bigger CSA in the ST and TFL. Kupa et al. [40] found CSA of muscles composed of primarily glycolytic fibers and muscle fiber type were directly proportional to initial and change in conduction velocity; therefore, greater initial frequencies reflect bigger glycolytic fibers, but these fibers’ velocities decline quicker. In the current study, 150NR ST and TFL muscles had a greater percentage of glycolytic fibers that were larger; however, 150NR ST SDH staining indicates these fibers possessed a greater oxidative metabolism. Therefore, 150NR ST muscles had smaller initial nMdPF than 0NR ST muscles because of the oxidative metabolism of those fibers. In contrast, 150NR TFL muscle-initial nMdPF did not drastically differ from 0NR TFL muscle, most likely because there was no difference in SDH staining intensity. During performance testing, the fact that 150NR ST and TFL fiber CSAs were larger could have caused smaller MdPF during performance testing. The larger-fiber CSA and smaller MdPF may be further contextualized by adaptation within the extracellular matrix which serves as a critical scaffolding for lateral force transmission; specifically, as fibers remodel and increase size, the matrix must adapt its mechanical properties to maintain structural integrity and prevent constraints on fiber contractility [41]. These results suggest fiber remodeling altered fiber morphology and muscle-specific conductive and metabolic characteristics were altered in a muscle-specific manner according to their propensity for shifting fiber. These muscle-specific alterations were further characterized by SDH intensity and capillary ratio. Fiber remodeling often involves reprogramming genes expressions to sustain performance, specifically activation of metabolic pathways that can drive a switch toward oxidative phenotypes which lead to increases in oxidation enzymes and mitochondrial biogenesis [42].

Nicotinamide adenine dinucleotide fuels oxidation and its reduced co-factors support oxidative phosphorylation and reactive oxygen species detoxification [9]; therefore, elevating NAD+ through NR supplementation can provide metabolic benefits to the skeletal muscle. In vitro, Canto et al. [14] report supplementing murine and human cell lines with 0 to 1000 µM NR increased intracellular NAD+ in a dose-dependent manner, with a maximum 66% increase when comparing 1000 relative to 0 µM. Airhart et al. [43] found an 83% rise in human blood NAD+ following escalating NR supplementation that reached 1000 mg twice daily for 8 d. Similarly, Conze et al. [22] observed human blood NAD+ levels increased after two-week supplementation with 100, 300, and 1000 mg per d by 22, 51 and 142%, respectively. Consistent with NR’s role as a potent NAD+ precursor, Xu et al. [44] reported that NAD+ levels in chick skeletal muscle rose in a dose-responsive manner, peaking at a 62% increase in the 1000 mM group relative to control or 0 mM NR. Skeletal muscle NAD+ quantification in barrows following NR supplementation remains absent from the literature. Although NR was administered to barrows by Hennesey et al. [21], muscle NAD+ levels went unreported. Similarly, protective effects observed in the Longissimus dorsi, specifically reduced apoptotic activation and maintained mitochondrial integrity, were attributed by Zhu et al. [45] to presumed NAD+ upregulation without direct measurement after NR supplementation. In the current study, NR increased overall NAD+ content by 157% after 7 d and by 492% after 14 d, and elevated NAD+ levels in BF, ST, and TFL by 224, 166 and 88% after 14 d, respectively. Therefore, increased skeletal muscle NAD+ level may have contributed to sustaining RMS activity through fatigue by supporting the metabolic resilience necessary for muscle fibers to preserve excitation–contraction coupling. Consistent with these findings, alterations in NAD+ availability were accompanied by changes in mitochondrial DNA expression.

Xu et al. [44] demonstrate NR increased chicken embryo mitochondrial DNA expression encoded genes such as cytochrome c oxidase subunit II, as well as NAD^+^-dependent regulators of mitochondrial biogenesis, including sirtuin-1. In mice, 400 mg/kg/day increased mitochondrial DNA expression and mitochondrial DNA encoded genes such as COXI and COXII by a 1.5 to 1.8-fold [46]. Signaling proteins or encoded genes were not evaluated in the present study; however, these findings support mitochondrial DNA expression use as an indicator of NR-induced mitochondrial adaptations. While Liu et al.’s [47] work does not quantify mitochondrial DNA within the skeletal muscle, the authors observed a 9.3-fold increase in mitochondrial DNA copy in preadipocytes after 12 g/day NR supplementation. In the current study, 150NR increased mitochondrial DNA expression on average across all muscles by 2.98-fold after 14 d NR supplementation, with greater-fold increase observed in the BF (3.97-fold). These finding align with previous reports by Xu et al. [44] and Hennesey et al. [21], who observed increases in mitochondrial DNA expression of 74% in hatched chicks and between 38 and 55% in finishing barrows following supplementation when injecting NR at 1000 mM and 45 mg/kg of body weight via drench, respectively. Although mitochondrial DNA was positively influenced by 150NR, Mckenna et al. [48] demonstrate mitochondria can function at different efficiencies even when total DNA content remains unchanged; therefore, mitochondrial efficiency was also measured.

The relationship between mitochondrial enzyme activity and performance is well established in humans and mice [49] and was explored in cattle regarding feed efficiency [26]; yet ETCI, ETCII and CS activities have not been quantified in barrows before and after muscle activity. Most porcine fatigue studies rely on behavioral or EMG data to estimate fatigue [22,24], leaving biochemical respiratory enzyme measurements unexplored. Because complexes I and II are essential for mitochondrial respiration and ATP production, increases in their activity indicate improved oxidative function [50]. In the current study, mitochondrial enzyme activities for ETCI and ETCII were normalized to specific enzyme activity rather than CS, which prioritizes functional density over oxidative capacity of the tissue. While 150NR supplementation increased relative ETCI and ETCII by 23 and 33%, respectively, the absence of differences when ETCI and ETCII activities were normalized to CS indicate respiratory capacity per mitochondrial unit remained unchanged. This pattern suggests fatigue resilience was driven by mitochondrial abundance expansion rather than by enhanced intrinsic individual respiratory complexes efficiency. While structural markers such as mitochondrial DNA and a 10% increase in CS activity suggest stimulated mitochondrial biogenesis, these static measures do not confirm enhanced oxidative capacity. Functional assessments such as high-resolution respirometry [45] or V-slope [51] analysis are required to validate metabolic clearance and central drive suppression [52]; however, such analyses are beyond the scope of the current study.

## 5. Conclusions

A large NR supplementation dose appears to induce multilayered neuromuscular and mitochondrial adaptations that enhanced EMG-measured fatigue resilience in barrows. While gross performance metrics were not affected, normalized RMS and MdPF data suggest NR supplementation altered initial recruitment efficiency and provided functional durability required to maintain fiber activation throughout the duration of physiological testing, suggesting a shift in neuromuscular strategy. At the cellular level, increased NAD+ availability, mitochondrial DNA expression, and elevated ETCI and ETCII activities suggest improved fatigue resilience driven by enhanced mitochondrial abundance. Consequently, NR may serve as a targeted nutritional strategy for pigs to better withstand the physical demands of loading, handling and transport. Accurate NR impact quantification on gross output requires performance testing protocols that are independent of behavioral influence which may otherwise mask important physiological improvements.

## Figures and Tables

**Table 1:** Effects of large-dose nicotinamide riboside supplementation on barrow performance test measurements.

	Treatment ^2^		SEM	*p*-Value
Item	0NR	150NR		
Performance test ^1^				
Lap, #	11.0	10.4	7.14	0.60
Distance, m	2830.1	2773.2	352.96	0.86
Time, s	777.6	798.0	122.84	0.83
Speed, m/s	3.9	3.8	14.80	0.60

^1^ Barrows were walked around a 270 m course until subjective exhaustion. Subjective exhaustion signs included stopping five times and displaying open mouth breathing, skin discoloration, abnormal vocalization, and muscle tremors. If barrows went down on front limbs, exhaustion was also designated. ^2^ Barrows were supplemented on 0 (**0NR**; *n* = 43) or 150 (**150NR**; *n* = 38) mg·kg body weight^−1^·d^−1^ nicotinamide riboside during the last 14 d of feeding.

**Table 2:** Effects of large-dose nicotinamide riboside supplementation on barrow immunohistochemical and histological muscle fiber characteristics across three muscles.

Item	Treatment ^1^						SEM	*p*-Value ^2^		
	*Bicep Femoris*		*Semitendinosus*		*Tensor Fasciae Latae*					
	0NR	150NR	0NR	150NR	0NR	150NR		MUS	TRT	MUS × TRT
Fiber type, %										
Type I	6.3 ^a^	5.4 ^b^	6.6 ^a^	5.1 ^b^	17.4 ^c^	12.5 ^d^	0.23	<0.01	<0.01	<0.01
Type IIA	32.2 ^a^	28.4 ^b^	26.9 ^c^	26.4 ^c^	14.7 ^d^	30.4 ^e^	0.29	<0.01	<0.01	<0.01
Type IIX	7.4 ^a^	10.4 ^b^	13.0 ^c^	9.5 ^d^	21.9 ^e^	9.8 ^b,d^	0.30	<0.01	<0.01	<0.01
Type IIB	54.1 ^a^	55.8 ^b^	53.5 ^a^	59.0 ^c^	45.9 ^d^	47.2 ^e^	0.59	<0.01	<0.01	<0.01
Cross-sectional area, µm^2^										
Type I	5242 ^a^	2632 ^b^	2605 ^b^	4990 ^a,d^	4036 ^c^	4749 ^d^	277.5	<0.01	0.19	<0.01
Type IIA	7426 ^a^	3992 ^b,d^	4352 ^a,d^	6449 ^c^	4217 ^d^	6585 ^c^	266.8	0.07	<0.01	<0.01
Type IIX	9970 ^a^	7718 ^b^	7856 ^b^	13,342 ^c^	8677 ^d^	7833 ^b^	328.9	<0.01	<0.01	<0.01
Type IIB	10,334 ^a^	7525 ^b^	6824 ^c^	11,696 ^d^	6635 ^c^	8985 ^e^	288.7	<0.01	<0.01	<0.01
SDH intensity ^3^, AU										
Type I	143.2 ^a^	128.6 ^b^	148.7 ^a^	138.9 ^a^	121.6 ^b^	128.6 ^b^	12.44	<0.01	0.19	0.08
Type IIA	153.2 ^a^	154.9 ^a,x^	167.7 ^b^	149.9 ^a^	143.6 ^a,y^	154.9 ^a^	9.10	0.09	0.65	<0.01
Type IIX	168.9 ^a^	180.4 ^b^	193.0 ^c^	168.6 ^a^	177.6 ^a,b^	175.8 ^a,b^	8.40	0.31	0.15	<0.01
Type IIB	188.5 ^a^	201.1 ^b,x^	214.1 ^c^	188.6 ^a^	195.4 ^a,b^	191.7 ^a,y^	6.53	0.06	0.06	<0.01
Capillary density ^4^										
Ratio	1.2 ^a^	1.30 ^b^	1.14 ^a,c^	1.12 ^c^	1.23 ^b^	1.14 ^a^	0.03	<0.01	0.73	0.02

^1^ Barrows were supplemented on 0 (**0NR**; n = 44) or 150 (**150NR**; n = 43) mg·kg body weight^−1^·d^−1^ nicotinamide riboside during the last 14 d of feeding. ^2^ Muscle (**MUS**) and treatment (**TRT**) main effects. ^3^ SDH = succinate dehydrogenase: 0 = most intense staining and 250 = least intense staining. AU = arbitrary units. ^4^ Capillary ratio was determined by dividing the total number of capillaries by the total number of muscle fibers. ^a,b,c,d,e^ Means within a row with different letters differ (*p* < 0.05). ^x,y^ Means within a row with different letters tend to differ (0.05 < *p* ≤ 0.10).

**Table 3:** Effects of large-dose nicotinamide riboside supplementation on citrate synthase (**CS**) activity from muscle biopsies collected on day 0 and 14.

Item	Treatments ^1^							*p*-Value ^2^			
	*Bicep Femoris*		*Semitendinosus*		*Tensor Fasciae Latae*						
	0NR	150NR	0NR	150NR	0NR	150NR	SEM	DAY	MUS	TRT	Interactions
nCS ^3^, μmol/min/g WW								0.03	<0.01	0.04	>0.13
D0	2.7	2.6	2.3	2.5	2.3	2.4	0.11				
D14	2.8	3.2	2.6	3.0	2.1	2.7	0.11				

^1^ Barrows were supplemented on 0 (**0NR**; *n* = 44) or 150 (**150NR**; *n* = 43) mg·kg body weight^−1^·d^−1^ nicotinamide riboside during the last 14 d of feeding. ^2^ Day (**DAY**), muscle (**MUS**), and treatment (**TRT**) main effects. ^3^ Data are normalized to d 0 0NR barrow CS activity within each muscle (**nCS**).

## Data Availability

The raw data supporting the conclusion of this article will be made available by the authors without undue reservation.

## Supplementary Materials

The following supporting information can be downloaded at: https://www.mdpi.com/article/10.3390/metabo16040261/s1, Table S1: Effects of large dose nicotinamide riboside supplementation on electron transport chain I and II (ETCI; ETCII) activity from muscle biopsies collected on day 0 and 14.

## Abbreviations

The following abbreviations are used in this manuscript:

DCPIP	2,6-Dichlorophenolindophenol
BF	Bicep femoris
CSA	Cross-sectional area
CS	Citrate synthase
DAY	Day main effect
DNA	Deoxyribonucleic acid
EMG	Electromyography
ETCI	Electron transport chain complex I
ETCII	Electron transport chain complex II
LARU	Large Animal Research Unit
MdPF	Median power frequency
MUS	Muscle main effect
NAD	Nicotinamide adenine dinucleotide
NADH	Nicotinamide adenine dinucleotide + hydrogen
NR	Nicotinamide riboside
nCS	Normalized citrate synthase
nETCI	Normalized electron transport complex I
nECTII	Normalized electron transport complex II
nMdPF	Normalized median power frequency
nNAD+	Normalized nicotinamide dinucleotide
nRMS	Normalized root mean square
PER	Period main effect
RMS	Root mean square
SDH	Succinate dehydrogenase
ST	Semitendinosus
TFL	Tensor fascia latae
TRT	Treatment main effect
