# Supplementary material for: Finishing Barrow Skeletal Muscle Performance and Fatigue Response to Large-Dose Nicotinamide Riboside Supplementation
Source: Metabolites. 2026 Apr 13;16(4):261. doi: 10.3390/metabo16040261 (PMC13117139; doi:10.3390/metabo16040261)
Supplement: Supplementary file 1 [file metabolites-16-00261-s001.zip › metabolites-4211288-supplementary.pdf]

Supplementary Table

S1. Effects of large dose nicotinamide riboside supplementation on electron transport chain I and II (**ETCI; ETCII**) activity from muscle biopsies collected on day 0 and 14

Item	Treatments[1]						SEM	*P*-value[2]			
	Bicep femoris		*Semitendinosus*		*Tensor fasciae latae*						
	0NR	150NR	0NR	150NR	0NR	150NR		DAY	MUS	TRT	Interactions
nETCI[3], μmol/min/g WW								<0.01	0.71	0.97	>0.33
D0	583.4	596.6	620.9	606.6	592.9	620.3	33.62				
D14	476.9	532.9	545.6	520.1	463.6	465.4	34.36				
nETCII[3], μmol/min/g WW								0.92	0.20	0.88	>0.19
D0	77.3	79.0	59.6	79.7	69.6	74.8	10.85				
D14	77.0	84.5	64.2	80.4	76.6	76.3	9.69				

[1]Barrows were supplemented 0 (**0NR; *n* = 44)** or 150 (**150NR; *n* = 43**) mg·kg body weight^{-1}·d^{-1} nicotinamide riboside during the last 14 d of feeding.

[2]Day (**DAY**), Muscle (**MUS**), and Treatment (**TRT**) main effects.

[3]Data were normalized to d 0 0NR barrow citrate synthase activity within each muscle (**nETCI; nETCII**).

products referred to in the content.
